# Development and initial evaluation of a behavioural intervention to support weight management for people with serious mental illness: an uncontrolled feasibility and acceptability study

**DOI:** 10.1186/s12888-023-04517-1

**Published:** 2023-03-01

**Authors:** Charlotte Lee, Felicity Waite, Carmen Piernas, Paul Aveyard

**Affiliations:** 1 Nuffield Department of Primary Care Health Sciences, Radcliffe Observatory Quarter, University of Oxford, Woodstock Road, Oxford, OX2 6GG UK; 2 Oxford Biomedical Research Centre, Oxford University Hospitals NHS Foundation Trust, Oxford, Oxfordshire UK; 3 Department of Psychiatry, University of Oxford, Warneford Hospital, Warneford Lane, Headington, Oxford, Oxfordshire OX3 7JX UK; 4 Oxford Health NHS Foundation Trust, Warneford Lane, Headington, Oxford, Oxfordshire OX3 7JX UK

**Keywords:** Mental illness, Schizophrenia, Bipolar, Weight, Intervention, Programme

## Abstract

**Background:**

The rates of obesity and associated health problems are higher in people with serious mental illness (SMI), such as schizophrenia and bipolar disorder, than the general population. A primary care referral to a behavioural weight management programme can be an effective intervention, but people with SMI have reported barriers to engaging with them and bespoke options are rarely provided in routine practice. It is possible that adjunct support addressing these specific barriers could help. Here we report the development, feasibility and acceptability of an intervention to improve uptake and engagement with a mainstream weight management programme for people with SMI.

**Methods:**

We worked with people with a lived-experience of SMI and used the person-based approach to develop the ‘Weight cHange for people with sErious mEntal iLlness’ (WHEEL) intervention. It comprised a referral to a mainstream weight management programme (WW®) to be attended once a week, in-person or online, for 12-weeks. The adjunct support comprised a one-off, online consultation called Meet Your Mentor and weekly, telephone or email Mentor Check Ins for 12-weeks. We assessed the feasibility of WHEEL through the number of programme and adjunct support sessions that the participants attended. We analysed the acceptability of WHEEL using a thematic analysis of qualitative interviews conducted at baseline and at 12-week follow-up. Our exploratory outcome of clinical effectiveness was self-reported weight at baseline and at end-of-programme.

**Results:**

Twenty participants were assessed for eligibility and 17 enrolled. All 17 participants attended Meet Your Mentor and one was lost to follow-up (94% retention). Nine out of 16 attended ≥50% of the weekly programme sessions and 12/16 attended ≥50% of the weekly check-ins. Participants reported in the interviews that the adjunct support helped to establish and maintain a therapeutic alliance. While some participants valued the in-person sessions, others reported that they preferred the online sessions because it removed a fear of social situations, which was a barrier for some participants. The mean change in self-reported weight was − 4·1 kg (SD: 3·2) at 12-weeks.

**Conclusions:**

A mainstream weight management programme augmented with brief and targeted education and low-intensity check-ins generated sufficient engagement and acceptability to warrant a future trial.

**Supplementary Information:**

The online version contains supplementary material available at 10.1186/s12888-023-04517-1.

## Background

Serious mental illness (SMI) refers to chronic illnesses like schizophrenia spectrum and bipolar disorder that are characterised by hearing, seeing and believing things not based on reality. People with SMI have an increased risk for poor physical health [1–3], with a 1·8 to three times higher risk of obesity (body mass index [BMI] > 30 kg/m^2^) than people without a diagnosis [4]. This contributes to a 3·8 higher risk for mortality from avoidable cardiovascular disease (CVD) before the age of 50 than the general population [5]. Meta-analyses of randomised controlled trials (RCTs) have shown antipsychotics, which are the mainstay treatment for most SMIs, cause weight gain; especially second-generation drugs like quetiapine, olanzapine and clozapine [6, 7]. Other meta-analyses have shown people with SMI to have higher dietary energy intake and lower levels of physical activity than the general population – behavioural patterns linked with obesity and CVD [8, 9].

Behavioural weight management programmes that support people to reduce their energy intake and increase their physical activity can be an effective intervention for obesity. In one RCT of 1267 participants, those assigned to the 12-week behavioural programme WW® (formally WeightWatchers®) lost 4·8 kg at one-year follow-up [10] and 2 kg at five-year follow-up [11]. Furthermore, evidence supports that these programmes are cost-effective in the short and long-term [10]. Accordingly, guidelines in the United Kingdom (UK) recommend a primary care referral for any patient with obesity to this type of mainstream programme, which are widely and freely available – at the point of prescription – in some countries’ health systems such as England [12].

Yet, even when referred, people with SMI may not engage with mainstream weight management programmes [13]. One trial of 1384 participants with SMI found the proportion of uptake was fewer than 2% compared with >50% in 12 studies included in a review of 30 studies in the general population [14, 15]. Meta-analyses of both survey data and qualitative accounts have reported physical (e.g., low energy), psychological (e.g., low confidence) and socio-ecological (e.g., lack of social support) barriers that can preclude access for people with SMI [16, 17]. While people from the general population may also experience these barriers [18, 19], people with SMI have reported additional issues that can prevent engagement. High levels of co-morbid anxious avoidance comparable to clinical agoraphobia are observed in people with SMI [20, 21]. This anxiety is likely to arise from and exacerbate low confidence, with high rates of social isolation and unhelpful eating behaviours that follow [22]. Interventions that target these barriers could therefore support people with SMI to benefit from current options for improved health.

A bespoke programme is one option to addressing these barriers for people with SMI. There is evidence that they can be modestly effective, with a meta-analysis of 41 RCTs across 4267 participants reporting 2·2 kg more weight loss than no or minimal intervention in follow-ups ranging from 8 to 52 weeks [23]. However, bespoke programmes are rarely part of routine care for people with SMI as a cost-effectiveness analysis has indicated these intensive programmes are not viable [24]. An alternative – arguably more sustainable – approach is to support people with SMI to engage with and benefit from mainstream programmes.

In a systematic review of 34 RCTs testing 36 behavioural weight management programmes in people with SMI, we found those that offered regular contact (e.g., weekly telephone calls), tools to support enactment (e.g., handouts) and tailored materials (e.g., shorter or repeated sessions) promoted engagement and were associated with clinical effectiveness [17]. Is it possible that providing these characteristics through adjunct support, alongside mainstream weight management, might enhance engagement with these programmes for people with SMI. However, the feasibility and acceptability of this approach is unknown. Here we report the development and initial evaluation of the ‘Weight cHange for people with sErious mEntal iLlness’ (WHEEL) intervention, which aimed to offer people with SMI adjunct support to access and engage with a mainstream weight management programme.

## Methods

### Intervention design

**Fig. 1:**
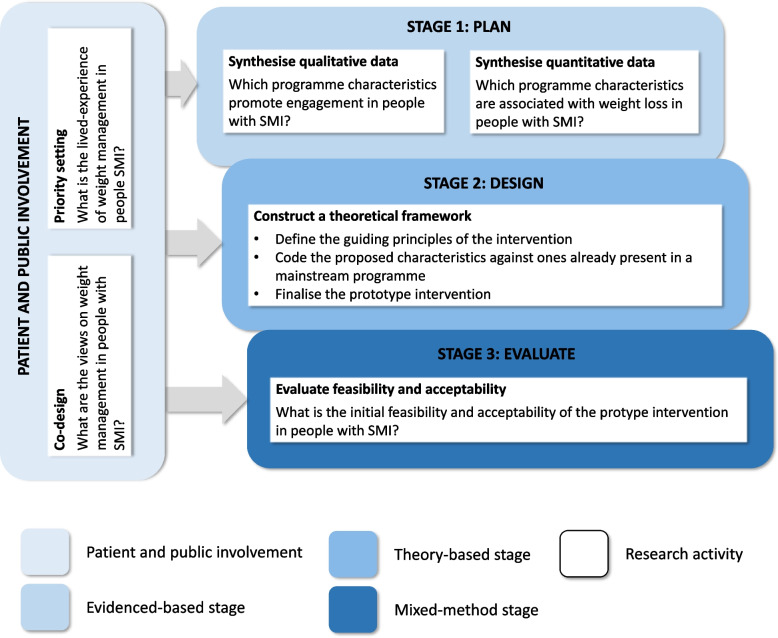
The three stages to develop and evaluate WHEEL

We worked with people with a lived-experience of SMI and used the person-based approach (PBA) to develop WHEEL. This helped us to create an intervention relevant for people with SMI and thereby improve the likelihood of it being effective [25]. The PBA has four stages: (1) plan, (2) design, (3) evaluate, and (4) implement and this paper covers the first three (see Fig. 1). Our patient and public involvement (PPI) and stages 1 and 2 (August 2020 to June 2021) are described in outline here and in full elsewhere [17]. Here we focus on stage 3 (June 2021 to July 2022). We followed the Template for Intervention Description and Replication (TiDieR) criteria [26] to describe the intervention and the Consolidated Criteria for Reporting Qualitative Studies (COREQ) criteria [27] to evaluate it (see Additional file 1: Supplementary material 1 and 2).

### Patient and public involvement (months 0–2)

We elicited views on the priority and direction of this work by consulting 12 people with a lived-experience of SMI. In total, we conducted five telephone interviews and two focus groups – one of four contributors, one of three contributors. Each consultation lasted 2 hours divided in two parts, with regular breaks to account for concentration difficulties. The first part covered a general discussion about their weight (e.g., the history of their weight gain, contributors to their weight gain and attempts and challenges to change). The second part covered their knowledge or experience of mainstream weight management programmes, with suggested improvements that we could enact in this study. All consultations were facilitated, audio-recorded and transcribed by the first author. Overall, the contributors recognised the need to manage their weight and were positive about the opportunity for support. There was some trepidation about mainstream weight management and we were cognisant of these concerns during our development. The contributors worked with us throughout the next stages to co-design the intervention (e.g., by reviewing themes during stage 1 and intervention handouts in stage 2).

### Stage 1: plan (synthesise qualitative data; months 0–13)

We systematically reviewed 20 qualitative studies to identify the barriers to engaging with behavioural weight management programmes and the programme characteristics that may help overcome them, as reported by people with SMI [17]. The nine barriers and corresponding characteristics are summarised in Additional file 1: Supplementary material 3.

### Stage 1: plan (synthesise quantitative data; months 0–13)

We systematically reviewed RCTs of bespoke weight management programmes for people with SMI to identify which characteristics were associated with weight loss [17]. We used a crisp-set qualitative comparative analysis (CsQCA) to establish causal relationships through systematic comparisons [28, 29]. Across 34 RCTs testing 36 programmes in 4305 people with SMI, those that offered interim booster support, supporting tools and tailored materials were more likely to result in greater weight loss than in the control group, compared with programmes that did not. The interventions resulted in more weight loss (mean = − 4.37 to + 1 kg at 6 weeks to 18 months follow-up) compared with controls (− 1.64 to + 3.08 kg).

### Stage 2: design (construct a theoretical framework; months 10–11)

We constructed a theoretical framework that was guided by the National Institute for Health and Care Excellence (NICE) recommended processes [30]. At this stage, we drew upon the results from stage 1 [17], relevant literature [31] and included key stakeholder feedback (i.e., from people with SMI and healthcare professionals).

#### Define the guiding principles

In the first phase we defined our guiding principles. This comprised the target behaviour(s); the hypothesised mechanism of change; the target barrier(s) that we had identified from stage 1 and considered necessary to address; the proposed characteristic(s) that we thought may help overcome each barrier; and the function of that characteristic (see Additional file 1: Supplementary material 4).

#### Code the proposed characteristics

In the second phase we coded the proposed characteristics against ones that were already present in the mainstream programme WW®. It is a commercially-provided, open-group that primary care clinicians can offer freely to patients in England with sessions usually attended once a week in-person for 12-weeks. This stage highlighted that WW® did not include some of the proposed characteristics. We therefore assessed which characteristics could be adjunctively provided alongside WW® (see Additional file 1: Supplementary material 5).

#### The prototype intervention

In the third phase, we constructed the prototype WHEEL intervention. It comprised a referral to WW® for 12-weeks to be attended in-person. The adjunct support was a single, 1·5 hour, online, one-to-one consultation called Meet Your Mentor. It aimed to address participants’ specific concerns and prime them with education on the modifiable contributors to weight gain for people with SMI. More specifically, Meet Your Mentor covered:

1. Your story and reasons for joining the intervention;
2. The causes of weight gain in people with SMI – recognising the weight gain effects of antipsychotics while emphasising self-regulation;
3. The challenges to losing weight in people with SMI – addressing barriers like negative self-beliefs, reasoning bias and social avoidance;
4. Activities to promote personalisation and boost engagement.

The mentor was a knowledgeable facilitator (i.e., a graduate-level psychologist; first author) who used a 17-page booklet, grounded in the principles of psychoeducation and cognitive behavioural therapy for psychosis (CBTp), that was reviewed by our PPI contributors. We offered regular breaks every 30 minutes. In addition, participants were given supporting tools (i.e., their personalised booklet), which they were encouraged to review in their own time.

After Meet Your Mentor*,* the mentor offered light touch practical support, activated the participants 12-week membership for the mainstream programme and found the date, time and location of their nearest in-person session. We felt that it was important to set clear expectations on their attendance at the first session of the mainstream programme to mitigate avoidance; however, we permitted moderate flexibility if participants were unable to attend that week. Thereafter the mentor provided 15-minute interim booster support over the telephone for 12-weeks to emphasise successes and achievements. We scheduled these Mentor Check Ins at a fixed day and time to increase the participants’ sense of accountability and support.

### Stage 3: evaluate (initial feasibility and acceptability; months 12–24)

#### Study design

We assessed the initial feasibility and acceptability of WHEEL in a single-arm, uncontrolled intervention study. The Medical Research Council (MRC) framework for developing complex interventions recommends identifying key uncertainties to be answered during feasibility testing [32]. Based on the literature [16, 17], we identified two key uncertainties to be answered in this study:

1. Feasibility defined as engagement with the mainstream programme and adjunct support, which we assessed using descriptive statistics (number [*n*, %] of Meet Your Mentor, programme sessions, and Mentor Check Ins attended);
2. Acceptability defined as positive responses for the mainstream programme and adjunct support, which we assessed using two qualitative interviews (one at the baseline and one at 12-week follow-up).

We also assessed the number (*n*, %) of participants retained at 12-weeks and self-reported weight at baseline and end-of-programme as exploratory outcomes.

#### Study sampling and recruitment

We advertised the study through PPI networks and used snowball sampling to recruit potentially eligible participants. We also advertised it through the McPin Foundation – a charity that exists to put the lived experience of people affected by mental health problems at the heart of research (www.mcpin.org). We provided interested participants with the information sheet prior to a telephone call to assess eligibility, obtain informed consent and collect demographic information.

##### Eligibility criteria

- Aged *≥*18 years;
- Given a primary diagnosis of SMI (e.g., schizophrenia, schizophreniform disorder, schizoaffective disorder, bipolar disorder, or depression with psychosis);
- Wanting to lose weight;
- Willing and able to join an in-person mainstream programme and discuss their experience of it in an audio-recorded interview;
- Willing and able to give informed consent for participation in the study.

We assessed the suitability of participants without overweight and obesity willing to join the study on a case-by-case basis. They were admitted to the study if they were taking medications known to cause weight gain and were concerned to limit that trajectory. All were keen to prevent excess weight gain and learn weight management strategies, especially given the weight gain trajectories in people with SMI [6, 7]. None had extensive weight loss during or at end-of-programme. Recruitment continued until we obtained sufficient data to address the research aims, which in this case were the indicative measures of feasibility and acceptability. Based on the sample size sometimes used in proof-of-concept and qualitative studies, we estimated 12 participants could be sufficient to meet this aim [33, 34].

### Procedure

Once participants finished their Meet Your Mentor session and agreed on their Mentor Check Ins dates, we sent them a personalised WHEEL booklet and voucher to access the mainstream programme. Attended their Meet Your Mentor and first programme sessions. This interview aimed to understand the acceptability of Meet Your Mentor; specifically, how it addressed their concerns and influenced their attendance at the first of 12 programme sessions. We invited participants to the second interview at 12-week follow-up. This interview aimed to understand the acceptability of the Mentor Check Ins; specifically, how it supported their engagement with the remaining 11 sessions (see Additional file 1: Supplementary material 6). The participants were remunerated £25 per hour per interview as UK guidelines recommend [35]. In line with the PBA, we analysed the qualitative interviews throughout programme delivery to modify WHEEL, which we recorded using the Table of Changes (TOC) method [25]. We assumed that no further responses after an enacted change was indicative of acceptance.

### Data collection

Demographic data were self-reported (including age, diagnosis, height and weight) at enrolment over the telephone. The mentor conducted all interviews over the telephone and she was trained in qualitative interviews. To mitigate respondent bias, the mentor prefaced questions, spotted inconsistencies to probe them and reassured participants that there were no right or wrong responses [36]. To mitigate interviewer bias, the mentor kept a reflexivity log to be cognisant of biases and debriefed with the study team on a fortnightly basis. The study team developed semi-structured topic guides for the two interviews (see Additional file 1: Supplementary material 7). Questions were initially open-ended, however, we modified our topic guides to include closed questions, which helped to scaffold conversations and break down difficult concepts. The mentor probed responses that she perceived as useful to the study aim. All interviews were audio-recorded, independently transcribed verbatim and checked by the mentor for accuracy against the recording.

### Data analysis

We used trial literature to categorise feasibility data as: ≤2 (low engagement), 3–5 (sporadic engagement), 6–10 (good engagement) and 10–12 (strong engagement) [37, 38]. For acceptability data, we used an inductive-deductive descriptive thematic analysis to code, categorise, identify and describe patterns in our transcriptions of the interview data [39]. This analysis followed four steps. First, we deductively developed a priori codes based on our PPI consultation. Second, we inductively added codes from each transcript. These codes were specific to the research aim. Third, codes were organised into sub-categories and then categories to produce top-level themes. Fourth, the study team discussed themes to reach a consensus agreement. We acted on Miles and Huberman’s recommendations to test and confirm our findings [40]. This meant we scrutinised the mentor’s reflexivity log during fortnightly discussions. We also invited participants’ feedback on the summary report of findings to ensure our interpretations remained close to their own accounts. Our ontological position was relativism and our epistemology was rooted in subjectivism. All data were managed in NVivo 1 software [41]. We present selected quotations under pseudonyms. We used a Cochrane publication to categorise the retention rate as: ≤49% (low), 50–79% (medium), and 80% + (high) [42].

## Results

### Demographic characteristics

We recruited participants between 12th August 2021 and 31st January 2022 and completed data collection on 30th April 2022. Once we met the recruitment target (*n* = 12), we purposefully recruited only males to ensure participants across the gender spectrum were equally represented in the data. Twenty participants were assessed for eligibility and 17 enrolled (i.e., three did not meet the inclusion criteria). Most participants were female (*n* = 13 [76%]), white (*n* = 8, [47%]) and living with schizophrenia spectrum disorder (*n* = 8 [47%]). The average age of participants was 48 years (range 29–70). Fourteen (82%) had baseline overweight and obesity.

### Feasibility

**Fig. 2:**
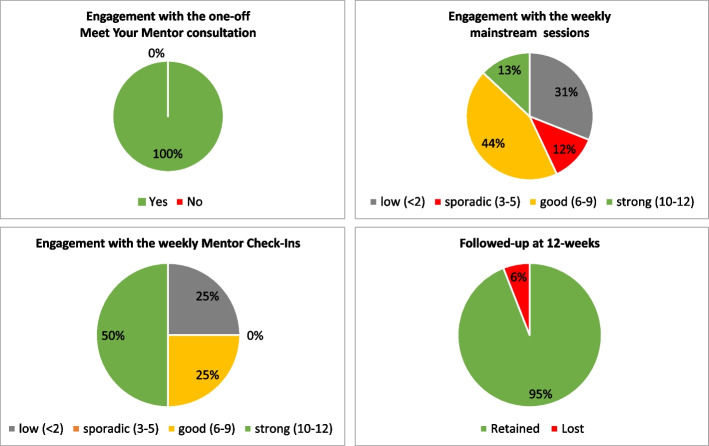
Descriptive data

We followed-up 16 out of 17 participants at 12-weeks (i.e., one lost without reason) and included them the analysis (95%, high retention). All 16 participants joined Meet Your Mentor (100%, strong engagement). For the programme sessions, five participants joined ≤2 (31%, low engagement); two participants joined 3–5 (13%, sporadic engagement); seven participants join 6–10 (44%, good engagement); and two joined 10–12 (13%, strong engagement). For the Mentor Check Ins, four participants joined ≤2 (25%, low engagement); no participants attended 3–5 check-ins (0%, sporadic engagement); four participants attend 6–10 (25%, good engagement); and 8 attended 10–12 check ins (50%, strong engagement) (see Fig. 2). The one participant lost without reason joined Meet Your Mentor and neither attended any subsequent programme sessions or responded to the Mentor Check Ins.

### Acceptability

We approached data saturation on some categories after 13 first interviews although we interviewed all 16 participants retained at 12-weeks, which confirmed that we did not need to construct new categories or themes. First interviews lasted on average 53 minutes (range: 32–75) and second interviews lasted 45 minutes (range: 9–95). We constructed seven themes centred on WHEEL’s acceptability, which we present according to the programme components. These themes focussed on how helpful or not the adjunct support was in accessing and engaging with the programme.

#### Meet your Mentor

##### Theme 1: establishing a therapeutic alliance

This theme considers the acceptability of the person delivering the adjunct support.

Participants welcomed the opportunity to disclose their concerns prior to starting the programme, with some expressing that the mentor established a psychologically safe space.

*I think, personally… the empathy, the kind of active listening [the mentor] had going on, I think that was really good. That was something I think most people want… for me [it] was quite beneficial because it kind of made me think about weight a little bit more. I think that people with mental health problems, we have a, we have a way of kind of ignoring things, especially things that make stress bad or make us not feel great.* (Abdel, first interview).

Participants reported that their weight gain was due to the side effects of antipsychotics, which left some of them feeling out of control and demotivated. Hearing the mentor reflect on their experiences in a clear and respectful manner validated some participants’ experiences and authenticated the credibility of the information that followed.

*So hearing… there are… a lot of people that do have this problem from this particular type of medication, was, was very reassuring I suppose because you know when it is being brushed off all the time by psychiatrists you do start to think, ‘Well is it just me then?’* (Fionee, second interview).

*I’m on medication that literally forces me to eat… it’s either that or I end up in a psychiatric unit, like, I’m sorry but I don’t have a choice* (Abdel, first interview).

##### Theme 2: value of the booklet

This theme describes the content of the booklet and if participants perceived it as useful or not in addressing their barriers to initiating the weight management programme.

The information in the booklet was novel for some participants. They reported that they felt optimistic about initiating a weight loss activity (i.e., either starting WW® or changing their food choices).

*I felt encouraged to start the Weight Watchers*®*, but I didn’t feel pressured*. (Fionee, second interview).

*I think what I took away from that as the most useful thing was about making the decision, having the power to make the decision at that particular time, when I’m about to eat something. I think that’s stuck with me*. (Denise, first interview).

Others valued the opportunity to share their own and hear others' experience of weight management in the context of SMI.

*I found it useful going through the booklet… not necessarily things useful to know, although that craving part was the biggest thing that stuck with me. But going through the booklet was good and just feeling that you know there’s obviously a lot of other people in the same situation and you’re not alone with it can really help.* (Fionee, second interview).

Two participants, Denise and Marcella, noted that the common challenges to losing weight in people with SMI (e.g., negative self-beliefs, reasoning bias and safety-seeking behaviours) were irrelevant to them. They both suggested tailoring the booklet to their specific concerns, which we enacted after Marcella (e.g., by asking participants which challenges were relevant to them before discussing it further).

##### Theme 3: a mentor that helped pinpoint specific barriers to joining

This theme describes the outcome of Meet Your Mentor and if it supported participants to initiate the programme.

Most participants expressed concern to be in an unfamiliar place or attend a social interaction. For some, it was because auditory-verbal hallucinations (e.g., critical or threatening voices) felt frightening, which negatively affected their desire to engage with other people. For others, it was fear or judgement or paranoid concerns about the potential harm from others.

… [The voices say, they say] *you can’t do anything right* (Matthew, second interview).

*It could be quite [er] hard just to introduce yourself… like a [er] completely new… environment… because you can feel people are staring at you, or, or you could feel people talking about you… So that is quite, yeah that’s quite, can be quite scary.* (Marcella, first interview)

A few participants spoke of how low self-esteem seemed to exacerbate their fears of being rejected, negatively judged or attacked by the group. Some participants also expressed that it was difficult to wake up and join a session because of the antipsychotic induced fatigue.

*As I have a mood disorder, low self-esteem is already an issue for me (as I presume for others in the group). I have no intention of being weighed in front of others or (a)nother. I know I am overweight and it causes me great distress and anxiety. I do not want to add to this. It is embarrassing to be weighed. A judgement is being made.* (Jane, email correspondence shared with permission)

Access to a mentor with whom they had established a therapeutic rapport allowed participants to share these concerns. This, in turn, opened opportunities for collaborative reflection (i.e., using if-then statements, reframing reasoning style).

*[Me and the mentor] talked in a very gentle way about the problem that I was having and it offered a kind of solution… that was such a long time ago and my, my life has changed a lot since then, that I don’t have those feelings anymore because it’s like [the mentor] addressed them in the first meeting that we had.* (Alice, second interview)

#### Mainstream weight management programme

##### Theme 4: acceptability of the in-person vs online programme

This theme centres around the acceptability of the mainstream programme, specifically the modality in which it is delivered.

We initially invited participants to join the in-person sessions. However, most participants expressed a strong desire to attend the online sessions, which we enacted in our TOC. That was because some felt able to manage worries more easily from a distance (e.g., reducing eye contact, staying mute and keeping the video off). They reported that these strategies reduced their concerns around being in unfamiliar social settings, though it prevented them from learning they could manage without such safety-seeking strategies.

*I’ve just gone into a room full of people that I didn’t get and thank god I could keep myself mute and nobody could see me. Where, where do I look [if I went in-person] when I say something that [others] just don’t agree with? You know, how do I manage my emotions?* (Tansi, first interview)

*[Online] is probably [er] better because you could [er] switch the, [um] the video off if [er] if you had [um] if you didn’t want to, to talk or to, to engage. And [er] that’s probably quite… useful for [er] for people with [er] with psychosis [er] because [er] the online features that let you just [er] hide yourself.* (Marcella first Interview)

The online modality enabled participants to still engage with the open-group. This was because it removed fear of social situations, which was a barrier for some participants.

*[The online session] was really good… because we talked about [um] I… just [typed] in the chat I said, ‘Look I’m just listening really and learning, here today, so I won’t say very much’.* (Tansi, first interview)

*So [online is] great and the things that I liked about [the coach] was that he spoke to everyone individually [using the one-to-one chat function].* (Abdel, first interview)

However, other participants expressed difficulties with using technology to access the online sessions. The frustration and rumination that followed sometimes led to unhelpful coping strategies (i.e., over-eating and binges)

*… for me it was the stress of trying to get on [the online session]… when you’ve got a mental health problem and you’re trying to get somewhere and you can’t get in and then you’re trying and trying, it just makes stress worse, which then exacerbates the condition, which meant that I ended up opening a box of Lindt chocolate and having most if it last night after, after the session because I just didn’t feel… well it was really, you know, I was just so angry… I messaged a friend and I said “I’m I don’t think I should do Weight Watchers at all… I’m going to give up.* (Abdel, first interview)

##### Theme 5: joining the programme amplified their sense of vulnerability

This theme focuses on the ongoing concerns reported by our participants that negatively affected the acceptability of the mainstream programme.

Whether it was the in-person or online programme session, some participants shared an ongoing sense of feeling either unsafe or that they did not fit in the group, which they attributed to their diagnosis of SMI. They described this as amplifying their sense of vulnerability, which meant they avoided attending the sessions.

*I wish I could think of another word other than I didn’t feel emotionally safe… because… I can’t afford to be putting myself in situations where I may feel a little bit vulnerable.* (Christie, first interview after they declined to join any more in-person sessions).

One participant shared concerns that the mainstream programme was not culturally appropriate to their needs, particularly the foods discussed in the session. This added to a sense of feeling pushed out from the group.

*People like myself, the people who still eat their own foods, who don’t really venture into westernised type meals and foods… [the programme] doesn’t really cater towards people like us and how do we make it more culturally competent and able so that people like myself who unwittingly have to gain weight because of the medication can then hopefully be able to manage our weight.* (Abdel, second interview)

#### Mentor check in

##### Theme 6: maintaining an interaction with the mentor

This theme circles back to theme 1 and discusses the continuity of care that the Mentor Check Ins provided.

Most participants described the check-ins as a chance to talk with the mentor about their weight loss, which they thought was lacking in the mainstream programme.

*I did really enjoy that side of it… because even though I did go to the workshops, you don’t always get the opportunity to speak when you’re there, and although you do have a check-in with the coach it’s, it’s still, it’s very different to having that conversation with [the mentor] each week… it was just more personal.* (Fionee, second interview)

This sense of protected time motivated participants to continue going to some of the sessions.

*I think mental health plays a big role … ‘cos sometimes your mental health can make something very small turn into something big task… but I I think for me one of the motivators was the fact that we had catch up calls… there was something about them that made me think ‘I’ve got to see this through, I can’t back out’. If I had joined on my own will and without the involvement of* [the mentor], *maybe after session one I probably would have given up, but* [the mentor] *helped me stay motivated.* (Hassan, second interview)

*I found them really good, really motivating to just, and encouraging, you know, to feel like I could look back on the achievements of the past week and have someone to share that with. If I was left to my own devices, I think it would have been a very different experience*. (Fionee, second interview)

#### Suggested improvements

##### Theme 7: define the nature of the mentorship

The participants offered recommendations for our future development, which this theme summarises.

All participants expressed that they valued the adjunct support, though it was notable that most also recommended a peer supporter with a lived-experience of SMI to address their ongoing feeling of isolation.

*It’s because a peer support worker can relate to you more easily and they’re cheaper ‘cos the NHS pay them less.* (Alice, second interview).

The content of the mentor check-ins needed refinement. One participant wanted a more structured check-in to account for their occasional disorganised thoughts and speech.

*No [the check-in was] really good when I could [answer]. I just wasn’t clear on what the check-in, I mean I know it was about how I… maybe a set of three questions [like] ‘how are you today’, ‘how has your week been’, ‘anything you’re looking forward to’ or ‘what was a great moment’. The reason I say that is because I’ve got fast acting bipolar so the thoughts are there, they just go really quickly through my mind.* (Tansi second interview).

The frequency and scheduling of the Mentor Check-Ins were also noted. Some participants felt that regular, scheduled, weekly check-ins were helpful and others wanted more flexibility.

*No I think once a week was good, and I enjoyed it being a phone call. I think it was about the right amount of time because it gave time to think to change.* (Fionee, second interview)

*There’s nothing wrong with the mentor, it’s just I thought if I can’t do a Thursday and I’m free on a Wednesday that week, it means at least I’m not missing a session.* (Hassan, second interview)

### Weight loss outcome

The mean self-reported weight change was − 4·1 kg (SD: 3·2) at 12-weeks from enrolment.

### Intervention changes

All proposed changes, and those subsequently enacted, were recorded using the TOC method (see Additional file 1: Supplementary material 8). The key changes were as follows. Meet Your Mentor: (1) tailor the booklet to each participant’s concern and strengthen the perceived relevance of the information provided; Meet Your Mentor: (2) extend from 15 to 30-minute telephone calls to allow for participant story telling; mainstream programme: (3) send email reminders to avoid participants’ forgetting their sessions.

## Discussion

### Main findings

This paper described how we combined person-, evidence- and theory-based approaches to develop, evaluate and iterate a mainstream weight management programme tailored for people with SMI. In our development, we highlight the number of stages, level of detail and the importance of continued refinement at the initial evaluation stage. In our evaluation, we have shown that a mainstream weight management programme bolstered with adjunct support generated sufficient feasibility and acceptability to run as an RCT.

### Strengths and limitations

We have clearly documented how we developed our intervention using established methods [32] and best practice guidance [26, 27]. This included finding and synthesising data using systematic reviews (e.g., of qualitative studies and RCTs) and our transparent decision-making on the augmented characteristics. We improved the prototype programme based on the participants’ direct experiences (e.g., from the qualitative interviews) and researcher observations, rather than hypothetical scenarios, which streamlined our development and allowed us to address challenges as they arose. However, we included a small sample size for a feasibility evaluation. We also made changes throughout delivery, which meant the interviews did not discuss a consistent intervention. This makes the number of participants discussing the same version of the intervention even smaller and this is likely to reduce reliability of the findings.

The mentor collected and analysed the data, which included qualitative interviews on the value of the mentorship. Since the mentor had built rapport with the participants, we may have we elicited responses that might not have been otherwise mentioned. However, this dual role is likely to have biased the data. Social desirability in qualitative methods is intractable and we prefaced questions, spotted inconsistencies to probe them and reassured participants that responses were not wrong to mitigate it [36]. To mitigate interviewer bias, we kept a reflexivity log to be cognisant of biases and debriefed with all other researchers on a fortnightly basis. We also followed published recommendations [40] (i.e., returning summary reports to the participants to ensure our interpretations were credible; triangulating evidence through comparisons with other published literature, reporting our work according to published guidelines), which strengthened the rigour of our findings. Participants also self-reported session attendance and weight change and one cannot rule out that social desirability also meant participants over-estimated their results, especially given reports of over-reporting in people with schizophrenia [43]. However, the reported weight loss resembles the results seen across meta-analyses suggesting the intervention likely led to weight loss [23].

The participants were ethnically diverse from across the UK and represent the intended people that could benefit from this support. Although most were female this reflects the demographics of people from the general population that take up mainstream programmes [10]. One cannot rule out that the intense contact, for the purpose of arranging the interviews and not the intervention itself, offered inadvertent support that the participant would not receive if it were offered as intended; although this is likely to be negligible. In terms of the intervention itself, WHEEL is designed to be opportunistically offered and delivered in primary care, however, there is no telling whether people with SMI would take up such a programme given the lack of empirical evidence.

### Comparison with the literature

A previous trial testing the effectiveness of a mainstream weight management programme in 11 people with schizophrenia and 11 matched controls reported a 3·3 kg weight loss in men only and a 50% follow-up rate at 12-weeks [44]. In another trial, 1384 participants with SMI were offered a choice between weight management interventions with fewer than 2% choosing the mainstream programme compared with 60% who chose the same programme with extra support from a health coach [14]. The data from these trials suggest that uptake and retention in mainstream programmes is likely to be low in people with SMI, perhaps due to unaddressed barriers to engagement, which we sought to investigate in this study.

We found that low-intensity support (i.e., a one-off initial consultation followed by weekly telephone or email exchanges from a mentor) is associated with 4·2 kg weight loss and a 94% follow-up rate, which is higher than previously reported in trials. These results are indicative and warrant further investigation but align with the results observed from other, more intensive, bespoke programmes. One RCT reported 54% of the 414 recruited participants had ‘good’ engagement with a bespoke programme and an 84% retention rate [24], compared with our 56% and 94%, respectively; though a subsequent cost-effectiveness analysis suggested that this type of support is unlikely to be cost-saving for health economies to provide [24]. The augmented elements of the intervention we provided are likely to be a feasible alternative of a potentially scalable augmented weight management programme.

### Implications for future research

The positive responses in our qualitative interviews largely centred on the weekly mentorship. In our development, we grounded this mentorship in the principles of CBTp (e.g., strong therapeutic alliance and collaboration exploration), which gave participants the opportunity to gather information that may lead them to update or change their beliefs. However, the modality (i.e., telephone or email), frequency (i.e., once a week or flexible) and content (i.e., open or structured discussion) needs further investigation. Our participants explicitly raised the potential value of working with peer supporters, with some evidence that they can lead to improved health outcomes in people with SMI [45]. Peer supporters are a part of a new workforce in some countries’ healthcare systems and they are likely to be more cost-effective than other healthcare professionals like psychologists. The adjunct support in our study provided by or in conjunction with peer supporters is an exciting area for future research.

## Conclusions

We describe the development and initial evaluation of adjunct support to improve uptake and engagement with a mainstream weight management programme for people with SMI. The results of this study potentially indicate that low-intensity mentorship in a randomised controlled trial might be feasible and acceptable for people with SMI.

## Supplementary Information

**Additional file 1: Supplementary material 1.** TiDieR checklist. **Supplementary material 2.** COREQ checklist. **Supplementary material 3.** Summary of results from stage 1 and 2. **Supplementary material 4.** The guiding principles of WHEEL. **Supplementary material 5.** Proposed characteristics coded against a mainstream weight management programme. **Supplementary 6.** A flow diagram of WHEEL. **Supplementary material 7.** Interview schedule. **Supplementary material 8.** Table of changes (TOC).

## Data Availability

The data generated or analysed during this study are available from the corresponding author on reasonable request.

## Abbreviations

CBTp: Cognitive behavioural therapy for psychosis
COREQ: Consolidated criteria for reporting qualitative research
MRC: Medical research council
NICE: National Institute for Health and Care Excellence
PPI: Patient and public involvement
PBA: Person-based approach
RCTs: Randomised controlled trials
SMI: Serious mental illness
TIDieR: Template for intervention description and replication
WHEEL: Weight cHange for people with sErious mEntal iLlness
