# Supplementary material for: Development and initial evaluation of a behavioural intervention to support weight management for people with serious mental illness: an uncontrolled feasibility and acceptability study
Source: BMC Psychiatry. 2023 Mar 1;23:130. doi: 10.1186/s12888-023-04517-1 (PMC9979581; doi:10.1186/s12888-023-04517-1)
Supplement: Supplementary file 1 — Additional file 1: Supplementary material 1. TiDieR checklist. Supplementary material 2. COREQ checklist. Supplementary material 3. Summary of results from stage 1 and 2. Supplementary material 4. The guiding principles of WHEEL. Supplementary material 5. Proposed characteristics coded against a mainstream weight management programme. Supplementary 6. A flow diagram of WHEEL. Supplementary material 7. Interview schedule. Supplementary material 8. Table of changes (TOC). [file 12888_2023_4517_MOESM1_ESM.pdf]

Title:

Development and initial evaluation of a behavioural intervention to support weight management for people with serious mental illness: an uncontrolled feasibility and acceptability study

Supplementary materials

Supplementary material 1. TiDieR checklist

Item number	Item and description	Reported	
		Primary paper (sub-heading): Prototype intervention	Other † (details)
**Brief Name**			
**1.**	Provide the name or a phrase that describes the intervention.	Primary paper (sub-heading): Prototype intervention	Abstract
**Why**			
**2.**	Describe any rationale, theory, or goal of the elements essential to the intervention.	Primary paper (sub-heading): Prototype intervention	N/A
**What**			
**3.**	Materials: Describe any physical or informational materials used in the intervention, including those provided to participants or used in intervention delivery or in training of intervention providers. Provide information on where the materials can be accessed (e.g. online appendix, URL).	Primary paper (sub-heading): Prototype intervention	N/A
**4.**	Procedures: Describe each of the procedures, activities, and/or processes used in the intervention, including any enabling or support activities.	Primary paper (sub-heading): Prototype intervention	N/A
**Who provided**			
**5.**	For each category of intervention provider (e.g. psychologist, nursing assistant), describe their expertise, background and any specific training given.	Primary paper (sub-heading): Prototype intervention	N/A
**How**			

**6.**	Describe the modes of delivery (e.g. face-to-face or by some other mechanism, such as internet or telephone) of the intervention and whether it was provided individually or in a group.	Primary paper (sub-heading): Prototype intervention	N/A
**Where**			
**7.**	Describe the type(s) of location(s) where the intervention occurred, including any necessary infrastructure or relevant features.	Primary paper (sub-heading): Prototype intervention	N/A
**When and how much**			
**8.**	Describe the number of times the intervention was delivered and over what period of time including the number of sessions, their schedule, and their duration, intensity or dose.	Primary paper (sub-heading): Prototype intervention	N/A
**Tailoring**			
**9.**	If the intervention was planned to be personalised, titrated or adapted, then describe what, why, when, and how.	Primary paper (sub-heading): Prototype intervention	Supplementary material 8. Table of Changes [TOC])
**Modifications**			
**10.**	If the intervention was modified during the course of the study, describe the changes (what, why, when, and how).	Primary paper (sub-heading): Procedure	Supplementary material 8. Table of Changes [TOC])
**How well**			
**11.**	Planned: If intervention adherence or fidelity was assessed, describe how and by whom, and if any strategies were used to maintain or improve fidelity, describe them.	N/A	N/A
**12.**	Actual: If intervention adherence or fidelity was assessed, describe the extent to which the intervention was delivered as planned.	N/A	N/A

† Hoffmann T, Glasziou P, Boutron I, et al. Better reporting of interventions: template for intervention description and replication (TIDieR) checklist and guide. *BMJ*. 2014; 348: g1687.

Supplementary material 2. COREQ checklist

Item number	Item	Description	Reported
**Domain 1: Research team and reflexivity**			
Personal Characteristics			
**1**	Interviewer/facilitator	Which author/s conducted the interview or focus group?	Primary paper (sub-heading): Data collection
**2**	Credentials	What were the researcher's credentials? E.g., PhD, MD	Primary paper (sub-heading): Prototype intervention
**3**	Occupation	What was their occupation at the time of the study?	Primary paper (sub-heading): Prototype intervention
**4**	Gender	Was the researcher male or female?	Primary paper (sub-heading): Data collection<br><br>See: Albury, C et al., Gender in the consolidated criteria for reporting qualitative research (COREQ) checklist, *International Journal for Quality in Health Care*, 33, 4, 2021. https://doi.org/10.1093/intqhc/mzab123
**5**	Experience and training	What experience or training did the researcher have?	Primary paper (sub-heading): Data collection
Relationship with participants			
**6**	Relationship established	Was a relationship established prior to study commencement?	Primary paper (sub-heading): Data collection
**7**	Participant knowledge of the interviewer	What did the participants know about the researcher? e.g. personal goals, reasons for doing the research	N/A
**8**	Interviewer characteristics	What characteristics were reported about the inter viewer/facilitator? e.g. Bias, assumptions, reasons and interests in the research topic	Primary paper (sub-heading): Data analysis
**Domain 2: study design**			
Theoretical framework			

**9.**	Methodological orientation and Theory	What methodological orientation was stated to underpin the study? e.g. grounded theory, discourse analysis, ethnography, phenomenology, content analysis	Primary paper (sub-heading): Data analysis
Participant selection			
**10.**	Sampling	How were participants selected? e.g. purposive, convenience, consecutive, snowball	Primary paper (sub-heading): Study sampling and recruitment
**11.**	Method of approach	How were participants approached? e.g. face-to-face, telephone, mail, email	Primary paper (sub-heading): Study sampling and recruitment
**12.**	Sample size	How many participants were in the study?	Primary paper (sub-heading): Demographic characteristics; Feasibility
**13.**	Non-participation	How many people refused to participate or dropped out? Reasons?	Primary paper (sub-heading): Feasibility
Setting			
**14.**	Setting of data collection	Where was the data collected? e.g. home, clinic, workplace	Primary paper (sub-heading): Data collection
**15.**	Presence of non-participants	Was anyone else present besides the participants and researchers?	N/A
**16.**	Description of sample	What are the important characteristics of the sample? e.g. demographic data, date	Primary paper (sub-heading): Demographic characteristics
Data collection			

**17.**	Interview guide	Were questions, prompts, guides provided by the authors? Was it pilot tested?	Primary paper (sub-heading): Data collection
**18.**	Repeat interviews	Were repeat inter views carried out? If yes, how many?	NA
**19.**	Audio/visual recording	Did the research use audio or visual recording to collect the data?	Primary paper (sub-heading): Data collection
**20.**	Field notes	Were field notes made during and/or after the interview or focus group?	Primary paper (sub-heading): Data collection
**21.**	Duration	What was the duration of the inter views or focus group?	Primary paper (sub-heading): Acceptability
**22.**	Data saturation	Was data saturation discussed?	Primary paper (sub-heading): Acceptability
**23.**	Transcripts returned	Were transcripts returned to participants for comment and/or correction?	NA
**Domain 3: analysis and findings**			
Data analysis			
**24.**	Number of data coders	How many data coders coded the data?	NA
**25.**	Description of the coding tree	Did authors provide a description of the coding tree?	NA

**26.**	Derivation of themes	Were themes identified in advance or derived from the data?	Primary paper (sub-heading): Data analysis
**27.**	Software	What software, if applicable, was used to manage the data?	Primary paper (sub-heading): Data analysis
**28.**	Participant checking	Did participants provide feedback on the findings?	Primary paper (sub-heading): Data analysis
Reporting			
**29.**	Quotations presented	Were participant quotations presented to illustrate the themes/findings? Was each quotation identified? e.g. participant number	Primary paper (sub-heading): Acceptability
**30.**	Data and findings consistent	Was there consistency between the data presented and the findings?	Primary paper (sub-heading): Acceptability
**31.**	Clarity of major themes	Were major themes clearly presented in the findings?	Primary paper (sub-heading): Acceptability
**32.**	Clarity of minor themes	Is there a description of diverse cases or discussion of minor themes?	Primary paper (sub-heading): Acceptability

† Tong A, Sainsbury P, Craig J, et al. Consolidated criteria for reporting qualitative research (COREQ): a 32-item checklist for interviews and focus groups. *International Journal for Quality in Health Care*. 2007; 19(6): 349 – 357.

Supplementary material 3. Summary of results from stage 1 and 2

**Barrier**	**Characteristics**		**Function**
Uncertainty on how antipsychotics affect weight, which can undermine psychological capability	1	Education on the specific contributors to weight gain for people with SMI	To improve knowledge and confidence
Low self-esteem attributed to negative self-beliefs and reasoning biases (e.g., confirmation bias, catastrophizing, all-or-nothing thinking), which can undermine motivation	2	Emphasis on successes and achievements	To reframe self-beliefs and reasoning style
Withdrawal and isolation due to the symptoms of SMI and societal stigma	3	A knowledgeable facilitator	To convey empathy, respect and meet the needs for rewarding social contact
	4	Peer support	
Fluctuating symptoms and medication side-effects, which can lead to varying motivation despite intentions to change	5	Interim booster support	To help translate intention into action
	6	Supporting tools	
Concentration difficulties due to psychotic experiences (e.g., hearing, seeing or believing things not based on reality)	7	Tailored materials like content written in plain and simple language and sessions that are shorter or repeated	To reduce cognitive burden and to promote accessibility
High levels of worry and social anxiety	8	Practical support	To reduce fears of travelling to unfamiliar places
N/A	9	The participants included in this review also welcomed incentives	

† Lee C, Piernas C, Stewart C, et al. Identifying effective characteristics of behavioral weight management interventions for people with serious mental illness: A systematic review with a qualitative comparative analysis. *Obesity Reviews*. 2022; 23(1): e13355.

Supplementary material 4. The guiding principles of WHEEL

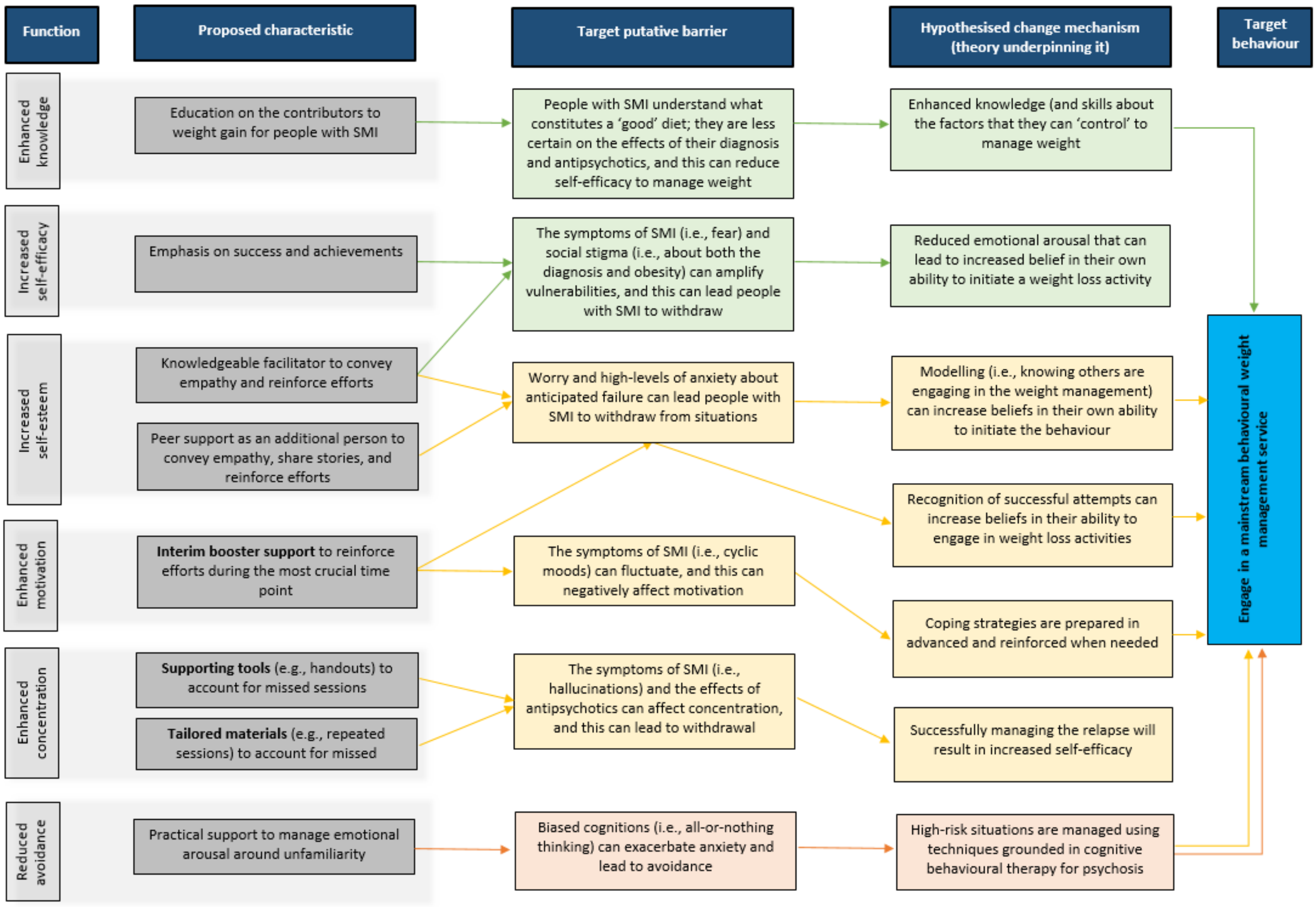

† The colour coding corresponds to the activity that was undertaken; the grey boxes denote either the programme function or characteristic; the green boxes denote an attitudinal barrier and hypothesised change mechanism; yellow boxes denote an emotional one; orange boxes a behavioural one; and the bright blue box denotes the target behaviour.

[b] Characteristics in bold denote the most effective characteristic identified from stage 1.

Supplementary material 5. Proposed characteristics coded against a mainstream weight management programme

Proposed characteristic		Corresponding characteristic in WW®	Decision to augment	Reason(s)
Knowledgeable facilitator:	As the first-person to initiate contact	WW**®** sessions are facilitated by a coach trained in weight loss and nutrition certified by the Association of Nutrition. They are also trained in providing one-to-one support and presentation skills.	✓ Include a knowledgeable facilitator	We judged that people with SMI might feel isolated and living in fear; it is important to establish psychological safety before joining a WW® session, which can be achieved through contact with a person knowledgeable about SMI
	To help review the barriers that preclude uptake and engagement; to encourage collaborative exploration of coping strategies using cognitive behavioural therapy for psychosis (CBTp)	WW**®** employs evidence-based strategies for weight loss including food journaling, regular group support, regular weight monitoring, attention to energy balance, learning nutritious and sustainable eating habits, and addressing the emotional component of weight loss using cognitive-restructuring techniques		We judged that the facilitator could review the barriers if (1) it overlaps with including 'Education on the specific contributors to weight gain for people with SMI*'; we judged that the facilitator could practise collaborative exploration if (2) it overlaps with including 'Interim booster support'
Education on the specific contributors to weight gain for people with SMI:	To recognise that antipsychotics can lead to weight gain and emphasise helpful cognitions that can support weight management	All WW® workshops provide education on nutritious and sustainable eating habits appliable	✓ Include targeted education	Specific education on the side-effects of antipsychotics is not given in WW®
Practical support:	Activate WW® account to manage emotional arousal	There is a helpline to call for help registering as a member. Since WW® offer open rolling meaning, attendees do not need to book a WW**®** session – they can find their nearest session a turn up	-- Offer 'light tough' practical support	We judged that sufficient practical support was available to join WW®. Offer minimal initial practical support if the attendee requests it only if (1) it overlaps with including a knowledgeable facilitator who can opportunistically do this
Emphasis on successes and achievements		All WW® workshops offer group-based opportunities to share 'wins' and 'loses'	✓ Offer additional opportunities to praise success and achievements	We judged that there were sufficient opportunities to praise wins and review losses in WW®. Offer additional opportunities to emphasise successes only (1) if it overlaps with including a knowledgeable facilitator who can opportunistically do this, and (2) it overlaps with interim booster support
Peer support:	As an additional person to accompany them to their first session	All WW® workshops advocate a friendly, supportive environment. WW® membership offers the 'Invite a Friend' option	✕ Do not offer peer support	We judged users may take an additional person to WW® at their own discretion, but that sufficient support would be given by the 'knowledgeable facilitator'
Interim booster support:	To maintain consistent support during the most crucial time points and remind users of their goals	Before each WW® meeting, coaches send a check-in message to see how users are getting on. Users also have access 24/7 support via our Online Coaches on our website	✓ Include interim booster support	We trialled out a 12-week WW® course during WHEEL's development and found coaches do not consistently send check-in messages. Furthermore, the 24/7 support puts the onus on the attendee to reach out for help during their most crucial times when they are precisely less likely to reach out

Tailored materials:<br><br>Repeat sessions to account for missed sessions	WW® offers open rolling groups meaning repeated sessions would be impractical.	✕ Do not include tailored materials	Users may discuss missed content at their next WW® workshop, but it will not be feasible to run repeated sessions
Supporting tools:<br><br>Handouts to account for missed sessions	WW® provide written curricula on healthy eating for weight loss and a verbal and written explanation of the program's 'point' system. Participants receive a calculator to measure foods, in 'points', simplifying use of the system.	-- Offer some supporting tools	All necessary material is provided by WW® meaning no additional tools are required. Offer handouts only if (1) targeted education is included

†WW® (formally WeightWatchers®) employs evidence-based strategies for weight loss including food journaling, regular group support, regular weight monitoring, attention to energy balance, learning nutritious and sustainable eating habits, and addressing the emotional component of weight loss using cognitive-restructuring techniques. Membership includes attendance at an unlimited number of meetings per week, although most members attend one per week. For the first 12 weeks of WW®, group leaders, who are successful lifetime members, provide written curricula on healthy eating for weight loss and a verbal and written explanation of the program's 'point' system. Participants receive a calculator to measure foods, in 'points', simplifying use of the system. Members are encouraged to exercise 20 minutes per day. Members are also able to access the on-line version of WW®, which includes a variety of weight loss resources including recipes and access to virtual workshop.

Supplementary 6. A flow diagram of WHEEL

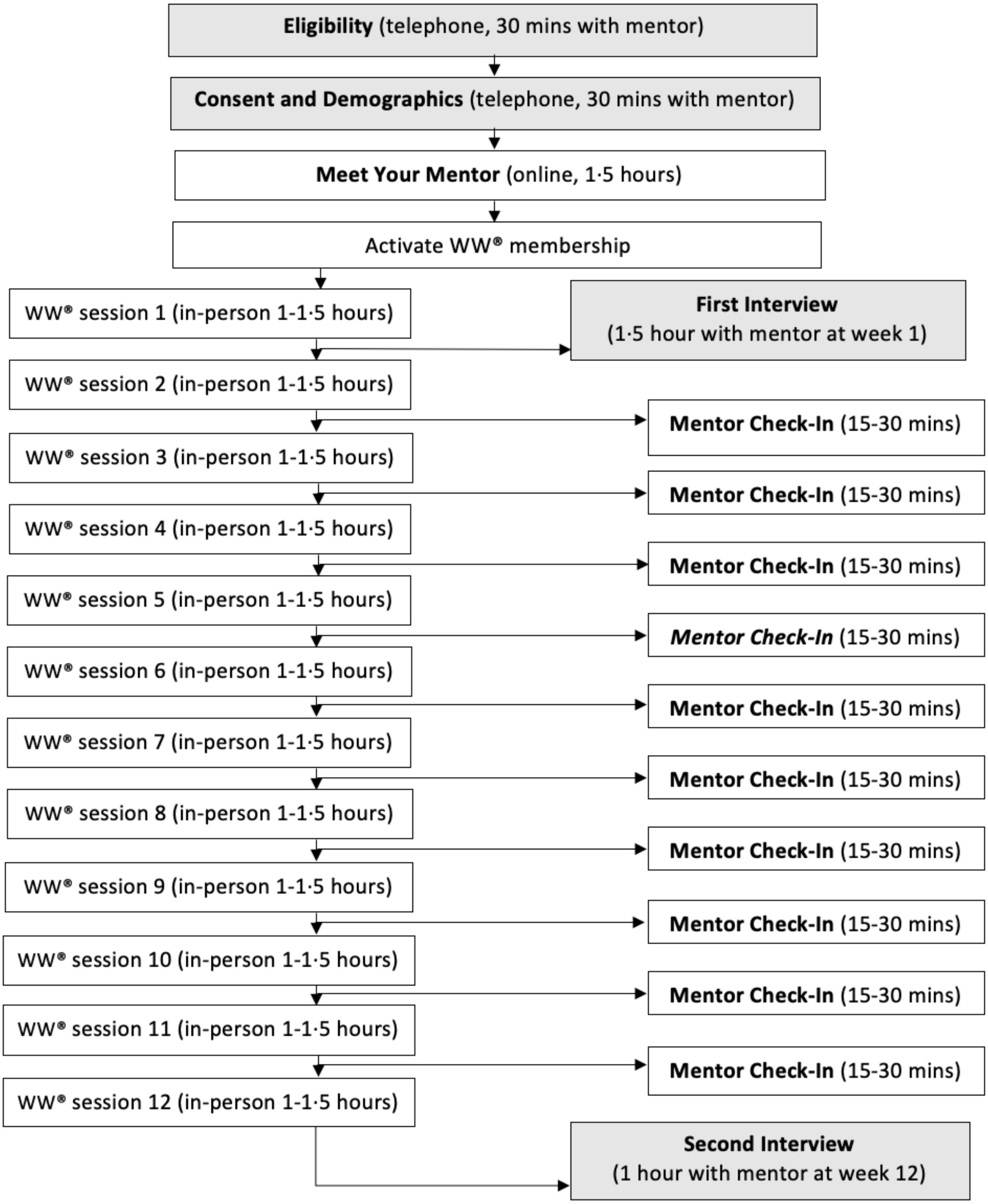

† The colour coding corresponds to the activity that was undertaken; the white boxes denote an intervention activity; the grey boxes denote a study activity.

Supplementary material 7. Interview schedule

**Questions**	**Prompts**
**First interview**	
Preamble	
1. Overall, how did you find that session?	If I asked you to name one thing that you liked about it and one thing that you didn't like, what would it be?
2. What was going through your mind as you joined this session?	Think about the night before and your travel journey the next day to the session – was there anything that you were excited or scared about?
3. How do you feel about the person delivering the session?	Think about the moment you entered the venue and joined the weigh-in queue – how did the coach seem? How were they throughout the rest of the session?
4. How do you feel about the other people in the session?	Think about the moment you sat down and waited with the other attendees for the session to start. What were your impressions about them?
5. Is there anything that helped or stopped you from going to the session and participating in the group?	Did it feel hard to go and then talk in the session? If so, why?
6. In what ways, if any, did Meet Your Mentor help you go to the session?	Was there anything that you found helpful or unhelpful about the session (e.g., the booklet)?
	How about the mentor. What are your thoughts about them?
	In what ways, if any, did it prepare you to join session?
7. Is there something else you would like to mention that you think may be important for me to know?	
**Second Interview**	
Preamble	
1. Think back to Meet Your Mentor. Can you tell me what you thought about it?	What do you remember about that session – is there anything that you'd like to add about it?
2. Tell me what you thought about the WW sessions?	What did you like about WW? What didn't you like about Weight Watchers?
	Have you done something different joining? Did you try to lose weight (e.g. count calories, reduce portions sizes)?

	In what ways, if any, did the session make you think differently about losing weight – does it seem easier or harder to lose weight?
	In what ways, if any, did the session make you think differently (e.g., feeling connected, isolated, or misunderstood)?
3. Over the past 12 weeks, we had regular check-ins. What did you think about them?	In what ways, if any, did the calls help you to lose weight?
	What did you like and not like about them?
	Is there anything you'd like us to do differently??
4. Imagine we were to run this study again. Is there anything you'd do differently?	
5. Is there something else you would like to mention that you think may be important for me to know?	

† Participant pseudonyms are given with each recommendation.

Supplementary material 8. Table of changes (TOC)

Aspect of intervention	Observation	Suggested change	Outcome
**Meet-Your-Mentor booklet**	Lead researcher in debrief with the research team after Fionee:<br><br>"Emphasise reasoning biases that are common in people with SMI"	Emphasise all the reasoning biases that are common in people with SMI and address them using principles of cognitive-behavioural therapy for psychosis (CBTp)	✓ Yes with immediate effect
	Denise, Meet Your Mentor; Marcella, Meet your Mentor:<br><br>"Not all biases are relevant to me"	Strengthen perceived relevance of the booklet by (1) asking participants if this bias sounds familiar to them, and (2) adding/removing examples when mailing their personalised copy of the booklet	✓ Yes after Marcella
**Meet Your Mentor**	Tansi, Second Interview:<br><br>"Change the name from 'Introductory Session' to 'Meet Your Mentor' to make the role of the lead researcher clearer"	NA	✓ Yes with immediate effect
	Abdel, Second Interview:<br><br>"I would like to have met my WW® coach in this session"	Invite WW® coach to the Meet-Your-Mentor session	✕ No this would be unfeasible
**Mainstream sessions**	Fionee, First Interview:<br><br>"Some people forget to go to their session and a reminder might be helpful"	Send an email reminder the day of their session including the time and venue location	✓ Yes after Fionee
	Sally, First Interview:<br><br>"I wasn't expecting to start WW® so soon (within 1 day of Meet-Your-Mentor) and felt unprepared"	Offer participants a one-week grace period to join their first session	✓ Yes after Sally
	Tansi, Mentor Check In:<br><br>"I wish I was brave enough to tell you earlier on that I would have preferred an online session"	Offer flexible choice on in-person or online session	✓ Yes but maintain expectation on the benefits of group-based in-person sessions to increase opportunities for positive/rewarding social interactions
**Mentor-Check-In**	Lead researcher in fortnightly debrief with the research team:<br><br>"Extend check ins from 15 to 30 minutes to allow more time for participants to share stories"	NA	✓ Yes at the discretion of the mentor
	Tansi, Second Interview<br><br>"I have fast acting bipolar so the thoughts are there, they just go really quickly through my mind"	Offer more structure to the check ins with three key questions only	✓ Yes after Tansi

† Participant pseudonyms are given with each recommendation.

End of supplementary file
